# Local translation and retrograde axonal transport of CREB regulates IL-6-induced nociceptive plasticity

**DOI:** 10.1186/1744-8069-10-45

**Published:** 2014-07-04

**Authors:** Ohannes K Melemedjian, Dipti V Tillu, Jamie K Moy, Marina N Asiedu, Edward K Mandell, Sourav Ghosh, Gregory Dussor, Theodore J Price

**Affiliations:** 1 Department of Pharmacology, The University of Arizona School of Medicine, Tucson, USA; 2 Department of Cellular and Molecular Medicine, The University of Arizona School of Medicine, Tucson, USA; 3 Bio5 Institute, Tucson, USA; 4 Graduate Interdisciplinary Program in Neuroscience, Tucson, USA; 5 The University of Texas at Dallas, School of Behavioral and Brain Sciences, Dallas, USA; 6 Department of Neurology, Yale School of Medicine, New Haven, USA; 7 School of Behavioral and Brain Sciences, University of Texas at Dallas, JO 4.212, 800 W Campbell Rd, Richardson, TX 75080, USA

## Abstract

Transcriptional regulation of genes by cyclic AMP response element binding protein (CREB) is essential for the maintenance of long-term memory. Moreover, retrograde axonal trafficking of CREB in response to nerve growth factor (NGF) is critical for the survival of developing primary sensory neurons. We have previously demonstrated that hindpaw injection of interleukin-6 (IL-6) induces mechanical hypersensitivity and hyperalgesic priming that is prevented by the local injection of protein synthesis inhibitors. However, proteins that are locally synthesized that might lead to this effect have not been identified. We hypothesized that retrograde axonal trafficking of nascently synthesized CREB might link local, activity-dependent translation to nociceptive plasticity. To test this hypothesis, we determined if IL-6 enhances the expression of CREB and if it subsequently undergoes retrograde axonal transport. IL-6 treatment of sensory neurons *in vitro* caused an increase in CREB protein and *in vivo* treatment evoked an increase in CREB in the sciatic nerve consistent with retrograde transport. Importantly, co-injection of IL-6 with the methionine analogue azido-homoalanine (AHA), to assess nascently synthesized proteins, revealed an increase in CREB containing AHA in the sciatic nerve 2 hrs post injection, indicating retrograde transport of nascently synthesized CREB. Behaviorally, blockade of retrograde transport by disruption of microtubules or inhibition of dynein or intrathecal injection of cAMP response element (CRE) consensus sequence DNA oligonucleotides, which act as decoys for CREB DNA binding, prevented the development of IL-6-induced mechanical hypersensitivity and hyperalgesic priming. Consistent with previous studies in inflammatory models, intraplantar IL-6 enhanced the expression of BDNF in dorsal root ganglion (DRG). This effect was blocked by inhibition of retrograde axonal transport and by intrathecal CRE oligonucleotides. Collectively, these findings point to a novel mechanism of axonal translation and retrograde trafficking linking locally-generated signals to long-term nociceptive sensitization.

## Background

Nociception, including nociceptive plasticity that frequently accompanies injury, serves a vital physiological function in vertebrates and invertebrates with complex nervous systems. It prompts an escape response from a source of real or potential tissue damage and plasticity in the nociceptive system guards against further damage by promoting a protective response during healing which is crucial for recuperation and survival [1]. However, these same plasticity mechanisms can lead to exaggerated nociceptive sensitization or a failure of resolution of hypersensitivity after tissue healing has completed its course, both of which may contribute to the development of chronic pain states [2]. The elucidation of molecular mechanisms that promote injury-induced nociceptive plasticity can provide insight into the development and possibly maintenance of chronic pain states [3]. Molecular mechanisms that are conserved across the animal kingdom may have particular salience for understanding how and why pain becomes chronic.

A potential, highly conserved mechanism of nociceptive plasticity is local translation of new proteins in response to injury [4-7]. In aplysia, crush injury to sensory neuron axons creates a sustained hyperexcitability in those same axons that is dependent on local translation [4]. Moreover, transplantation of axoplasm from the site of an injured aplysia axon to an uninjured one creates a state of enhanced excitability matching that produced by injury. Interestingly, this appears to involve a local change in a protein that then undergoes retrograde transport potentially leading to a phenotypic change in the axon based on a transcriptional change [8]. Although the identity of the protein involved in this local change has not been formally elucidated, the time course of the effect is consistent with a locally translated protein. We, and others, have provided compelling evidence for local translation as an important feature in the sensitivity of mammalian nociceptors and their plasticity in response to injury [4-7]. Here the mechanistic target of rapamycin complex 1 (mTORC1) and extracellular signal regulated kinase (ERK)/eukaryotic initiation factor 4E (eIF4E) signaling pathways play a key role linking extracellular inflammatory factors to local changes in gene expression in nociceptor axons at the level of translation [6,9,10]. Having said this, proteins that are synthesized locally in response to factors that promote pain plasticity have not been identified.

“Hyperalgesic priming” models use an initial insult to produce plasticity in the nociceptive system that manifests as an enhanced propensity to a long-lasting pain state with a subsequent, normally sub-threshold, stimulus. Hyperalgesic priming serves as an important model for investigation of pain plasticity mechanisms because it provides a clear experimental platform for the investigation of mechanisms involved in the transition to a chronic pain state [2,11,12]. For instance, in mice and rats a single injection of interleukin-6 (IL-6) causes a transient mechanical hypersensitivity that resolves within 3 days. Importantly, in these primed animals, after they no longer show an overt nociceptive hypersensitivity, a normally sub-threshold injection of prostaglandin E2 (PGE_2_) causes a long lasting reinstatement of mechanical hypersensitivity [6,12,13]. The initiation of this priming event is blocked by inhibition of translation at the site of the primary insult [6] or, during the maintenance phase, by sequestration of spinal brain derived neurotropic factor (BDNF) [14] providing a possible link between changes in local gene expression within the paw and longer-term alterations in BDNF expression in dorsal root ganglion (DRG) neurons. This notion is consistent with findings in invertebrate axons in response to injury [4,8] potentially pointing to a conserved role for local translation of retrograde signaling factors in nociceptive plasticity. A candidate for this nascently synthesized retrograde signaling molecule is the transcription factor cAMP-responsive element (CRE)-binding protein (CREB). Significantly, CREB has been identified as a nascently synthesized protein within the axons of developing DRG neurons in response to nerve growth factor (NGF) where it is then retrogradely transported to the nucleus to regulate gene expression critical for the early survival of these neurons [15]. Based on this, we hypothesized that IL-6-mediated mechanical hypersensitivity and hyperalgesic priming may be dependent on the local synthesis and retrograde trafficking of CREB.

In this study we provide evidence that CREB is locally translated in response to intraplantar administration of IL-6. Furthermore, we detail observations consistent with retrograde transport of this nascently synthesized CREB linking local changes in gene expression on the level of translation to enhanced BDNF expression in DRG neurons. Our findings provide evidence for a conserved mechanism of nociceptive plasticity.

## Results

### IL-6 induces the expression of CREB in vivo and in vitro

**Figure 1:**
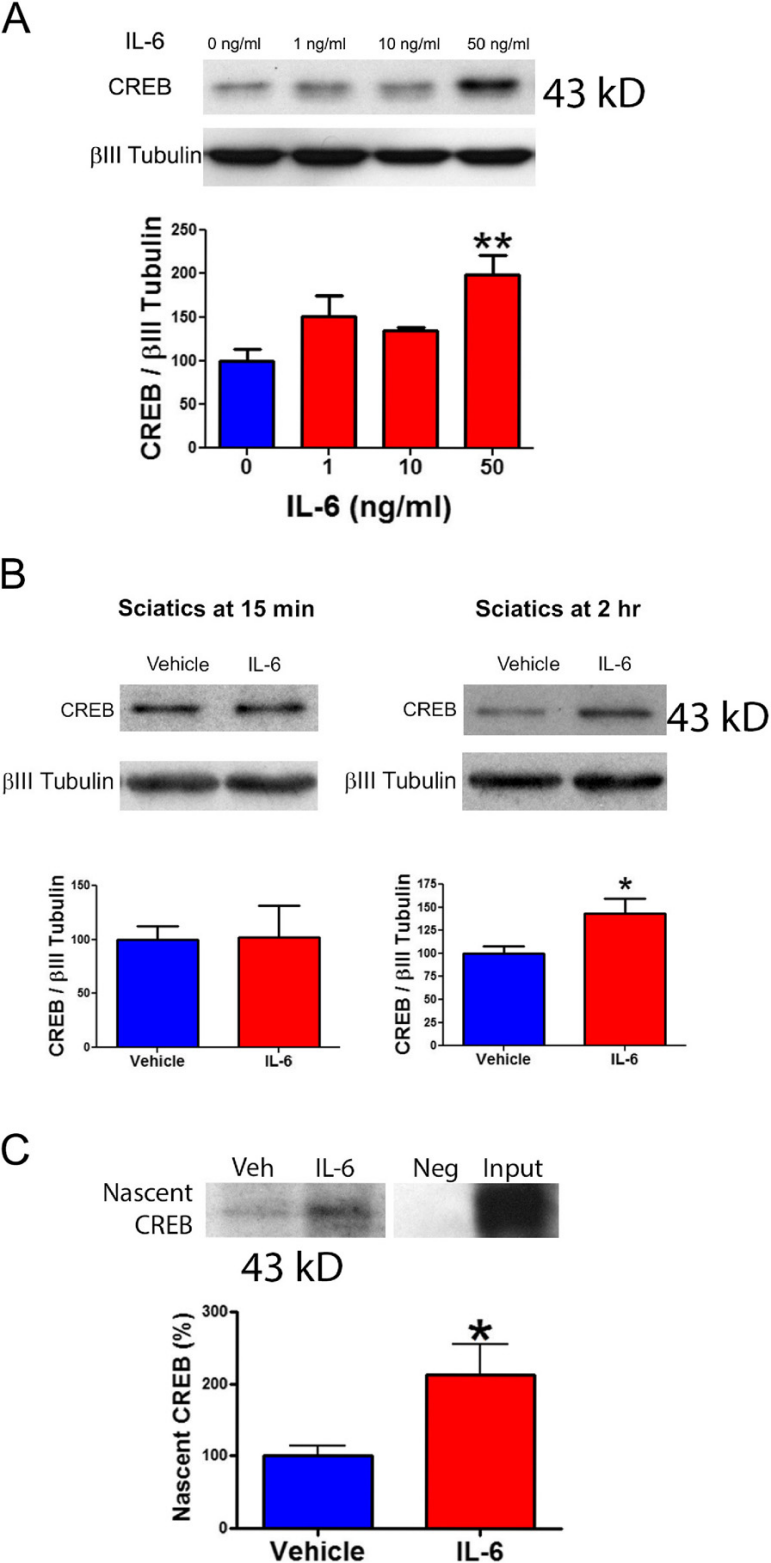
**IL-6 induces CREB synthesis in vitro and in vivo. A)** Western blot and quantification of CREB following 15 min treatment of DRG cultures with escalating concentrations of IL-6. N = 8 independent culture wells per condition. **B)** Western blot and quantification of CREB in the ipsilateral sciatic nerve following intraplantar (IPL) injection of IL-6 (0.1 ng) or vehicle. CREB protein is enhanced in the sciatic nerve 2 hrs post-injection but not at 15 min following treatment. N = 6 mice per condition. **C)** Western blot and quantification for nascently synthesized CREB retrogradely transported to the sciatic nerve. Retrograde axonal trafficking of nascently synthesized CREB was determined by co-injecting IL-6 (0.1 ng) or vehicle with the methionine analogue AHA into the paw and measuring, from the sciatic nerve, the amount of CREB with incorporated AHA, 2 hrs post injection. The input lane contains lysate from sciatic nerve that did not undergo immunoprecipitation and is labeled with biotin-avidin on incorporated AHA. N = 6 mice per condition. * = p < 0.05 and ** = p < 0.01.

The transcription factor CREB is synthesized within axons of developing DRG neurons in an NGF-dependent fashion and transported back to the neuronal nucleus where it influences transcription of genes regulating the survival of DRG neurons [15]. Local translation plays a key role in the sensitization of mammalian nociceptors, and is involved in the initiation and maintenance of hyperalgesic priming. Local translation is likewise linked to hypersensitivity arising in invertebrate sensory neurons after crush injury. We therefore hypothesized that local translation of CREB may link local translation of a transcription factor to IL-6-induced nociceptive plasticity. We have previously demonstrated that IL-6 activates the ERK/eIF4E pathway leading to enhanced translation of proteins in the axons of primary sensory neurons. Moreover, IL-6 induced mechanical hypersensitivity and subsequent PGE_2_-induced precipitation of hyperalgesic priming is blocked by inhibition of translation at the site of IL-6 injection [6]. As a first test of our hypothesis we asked if IL-6 induces a rapid change in CREB expression in DRG neurons. Treatment of DRG neurons in culture with IL-6 (50 ng/mL) for 15 minutes caused increased expression of CREB (Figure 1A) consistent with the time and concentration dependent activation of ERK and eIF4E phosphorylation we have determined previously.This finding suggests that DRG neurons can synthesize CREB in response to IL-6. In order to determine if an IL-6-mediated synthesis of CREB in DRG neurons can lead to retrograde transport, we injected IL-6 (0.1 ng) or vehicle into the paws of mice and collected the sciatic nerves at 15 min (a time point too rapid for retrograde transport) or 2 hrs (a time point where maximal retrograde transport would be predicted based on standard retrograde transport rates and the distance from the paw where the sciatic nerve was removed) later. As expected, at 15 min post injection, IL-6 did not induce an increase in the levels of CREB within the sciatic nerve (Figure 1B). However, 2 hrs following i.pl. injection of IL-6 we observed a significant increase in levels of CREB in the sciatic nerve (Figure 1B). These data suggest that locally synthesized CREB in the axons of DRG neurons in the paws of mice that were treated with IL-6 undergo retrograde transport into to the sciatic nerve. We next sought to demonstrate that this IL-6-induced CREB translocation to the sciatic nerve was nascently synthesized at the site of IL-6 injection. To this end we co-injected IL-6 with the methionine analogue AHA along with vehicle or IL-6 and sciatic nerves were dissected after 2 hrs. In order to detect AHA incorporation into nascently synthesized CREB we immunoprecipitated CREB and used “click chemistry” to label AHA with biotin, which was subsequently detected by Western blotting. Using this method, we observed an IL-6-dependent increase in nascently synthesized CREB in the sciatic nerve 2 hrs following treatment (Figure 1C). Collectively, these data provide evidence that IL-6 stimulates the local translation of CREB in axons of DRG neurons, which then undergoes retrograde transport into the sciatic nerve.

### Axonal retrograde transport is critical for the initiation and maintenance of IL-6-mediated hyperalgesic priming

**Figure 2:**
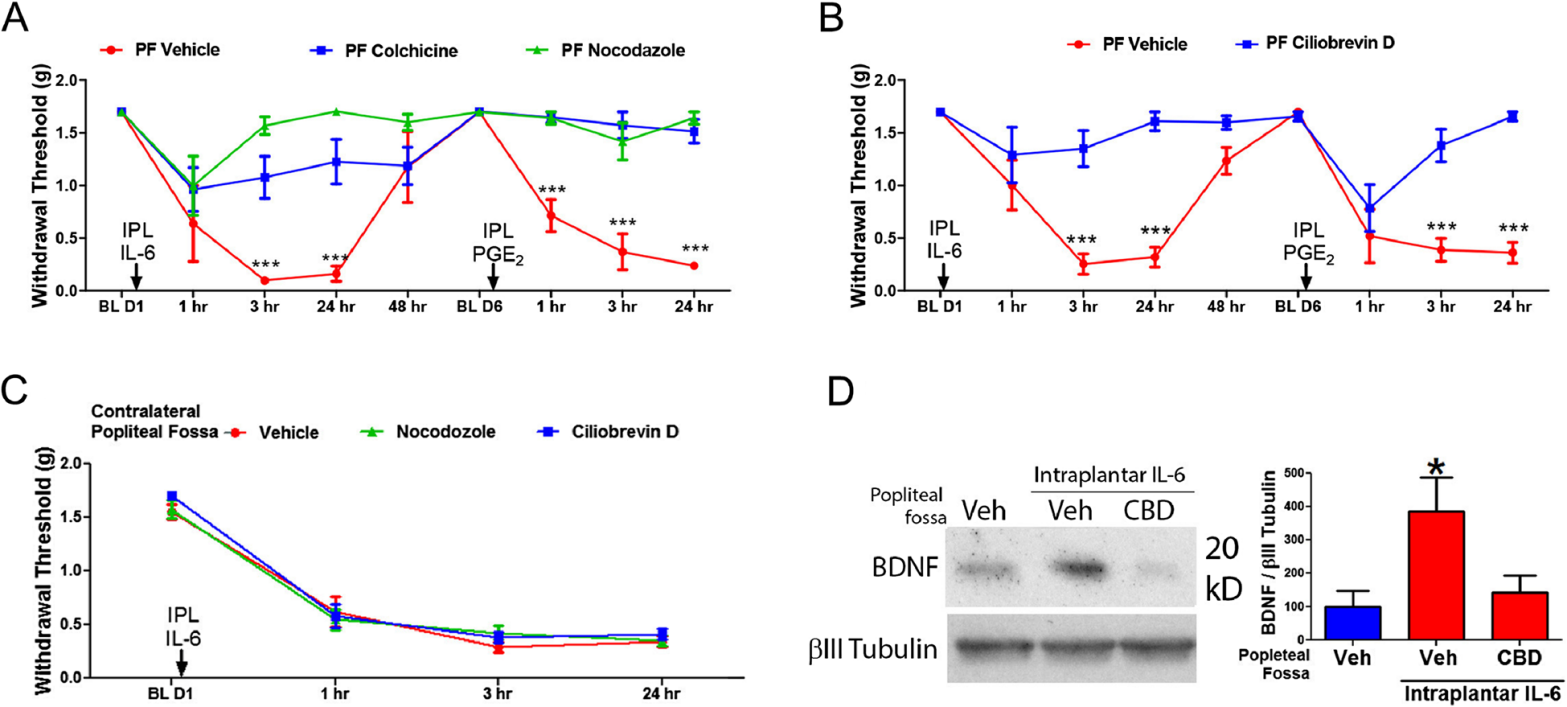
**Disruption of axonal trafficking in the sciatic nerve prevents IL-6-mediated nociceptive plasticity. A)** Disruption of microtubules with the injection of colchicine (100 μg) or nocodazole (10 μg) into the popliteal fossa (PF) of mice prevented the development of mechanical hypersensitivity and hyperalgesic priming caused by intraplantar injection of IL-6 (0.1 ng). N = 6 mice per condition. **B)** Popliteal fossa injection of the cytoplasmic dynein inhibitor ciliobrevin D (10 μg) prevented the development of mechanical hypersensitivity and hyperalgesic priming caused by Intraplantar injection of IL-6 (0.1 ng). N = 6 mice per condition. **C)** Right popliteal fossa injection of ciliobrevin D or nocodazole (both at 10 μg) fails to disrupt the effect of IL-6 given into the left hindpaw. N = 6 mice per condition **D)** Popliteal fossa injection of ciliobrevin D (CBD, 10 μg) at the time of intraplantar injection of IL6 prevented the enhancement of BDNF expression in the L4-L6 DRGs of mice 3 hrs following injections. N = 6 mice per condition. * = p < 0.05 and *** = p < 0.001.

Our hypothesis posits that translation links local signaling events to long-term changes in nociceptors via retrograde transport of a locally synthesized transcription factor. Hence, we next sought to determine the role of retrograde axonal transport in the development and maintenance of hyperalgesic priming. To this end, we used colchicine and nocodazole, which disrupt the polymerization of tubulin monomers to form microtubules, therefore inhibiting fast axonal anterograde and retrograde transport. We coupled the intraplantar injection IL-6 with the injection of vehicle, colchicine (100 μg) or nocodazole (10 μg) [8] in the popliteal fossa of mice. The popliteal fossa is next to the sciatic nerve and can provide a reservoir for drug to bathe the sciatic nerve following appropriate injection. Injection of vehicle into the popliteal fossa had no effect on the initiation or maintenance of IL-6 induced hyperalgesic priming (Figure 2A). However, popliteal fossa injection of either colchicine or nocodazole prevented IL-6-mediated mechanical hypersensitivity and hyperalgesic priming to subsequent PGE_2_ treatment (Figure 2A). In order to provide a more direct link to retrograde axonal transport as a critical mediator of IL-6-mediated mechanical hypersensitivity and hyperalgesic priming we used a selective inhibitor for cytoplasmic dynein, ciliobrevin D. Ciliobrevin D blocks dynein-dependent microtubule gliding and ATPase activity while sparing kinesin dependent cellular trafficking therefore selectively affecting retrograde transport [16]. Popliteal fossa injection of ciliobrevin D (10 μg) prevented both IL-6-induced mechanical hypersensitivity and hyperalgesic priming (Figure 2B). To assess whether the effects of nocodozole or ciliobrevin D could be attributable to systemic effects we injected these compounds into the contralateral popliteal fossa at the same dose at the time of IL-6 injection. As opposed to ipsilateral injections, neither compound had an effect on IL-6-induced mechanical hypersensitivity at any time point when given contralaterally (Figure 2C). These results strongly suggest that retrograde axonal transport is necessary for IL-6-mediated nociceptive plasticity.

### IL-6-mediated changes in DRG BDNF expression are linked to retrograde CREB signaling

**Figure 3:**
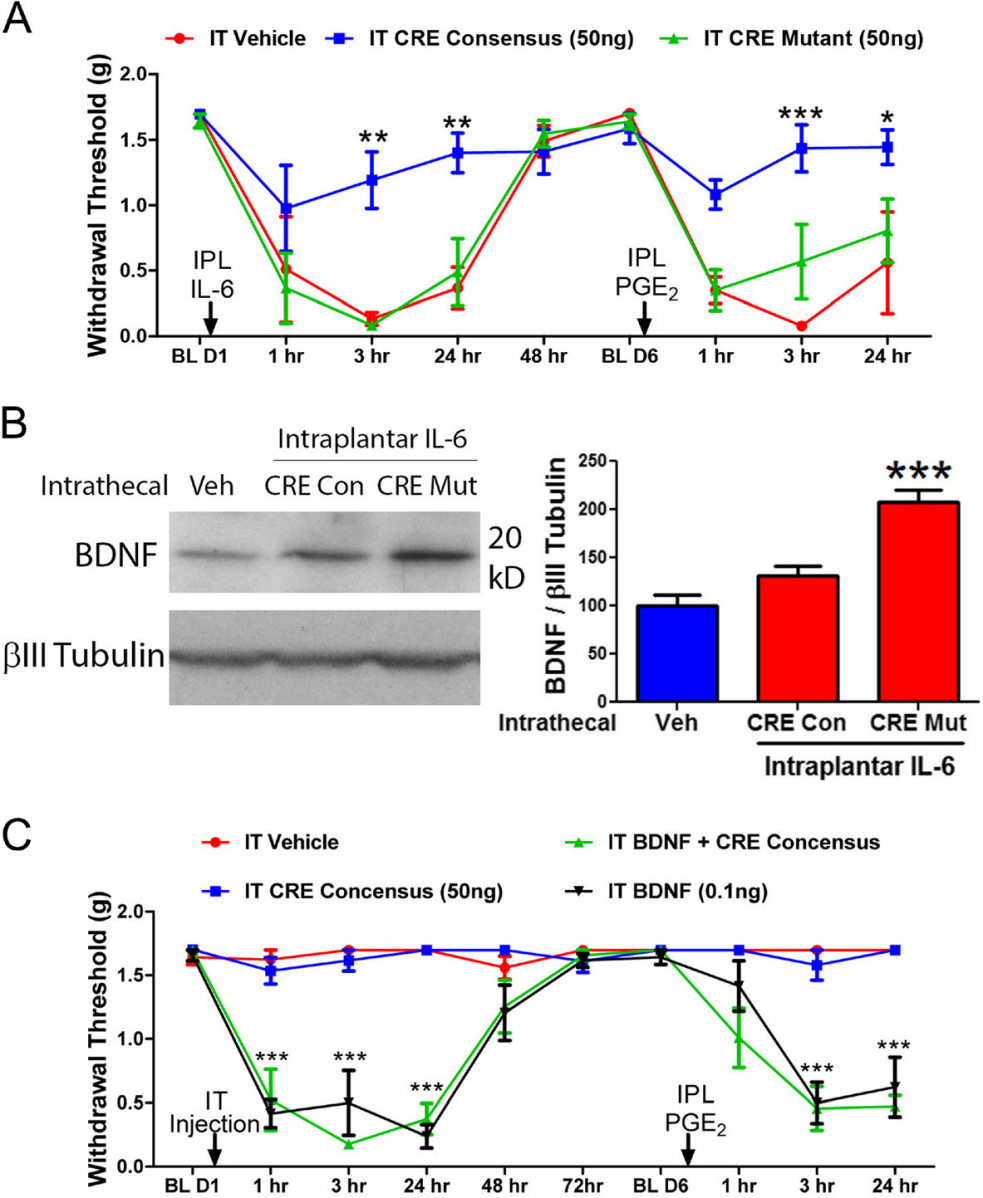
**Disruption of CREB action in the DRG attenuates IL-6-induced nociceptive plasticity. A)** Intrathecal injection of cAMP response element (CRE) consensus sequence DNA oligonucleotides at the same time as intraplantar IL-6, prevented the development of IL-6-induced mechanical hypersensitivity and hyperalgesic priming. Mutant CRE oligos were not effective. N = 6 mice per condition. **B)** Intrathecal injection of CRE consensus sequence DNA oligonucleotide (CRE con) at the same time as intraplantar IL-6, prevented enhancement of BDNF expression in L4-L6 DRGs 3 hours following injections. N = 6 mice per condition. **C)** Intrathecal injection of BDNF (0.1 ng) mechanical hypersensitivity and hyperalgesic priming. Co-injection of CRE consensus sequence DNA oligonucleotides with BDNF did not affect mechanical hypersensitivity and hyperalgesic priming. N = 6 mice per condition. * = p < 0.05, ** = p < 0.01 and *** = p < 0.001.

BDNF is released by nociceptors in response to noxious stimulation and has a profound effect on post-synaptic dorsal horn neurons. BDNF expression shows remarkable plasticity in the DRG following inflammation and BDNF expression in nociceptors is critical for inflammatory injury induced nociceptive plasticity. We have recently shown that IL-6-induced mechanical hypersensitivity and hyperalgesic priming are dependent on BDNF/trkB signaling [14]. Interestingly, IL-6-induced hyperalgesic priming can be disrupted at any time by blocking BDNF/trkB signaling suggesting that IL-6 mediated priming fundamentally changes BDNF/trkB signaling between DRG and dorsal horn neurons. How is this regulated? CREB is known to regulate the expression of BDNF in neurons [17], therefore, we determined whether expression of BDNF is increased following i.pl. injection of IL-6 and whether the blockade of retrograde axonal transport would prevent this effect. We again used popliteal fossa injections of ciliobrevin D at the time of i.pl. injections of IL-6 and measured the expression of BDNF in the dorsal root ganglia 3 hours post-injection (a time point at which CREB retrogradely transported from the paw should reach the DRG). We determined that intraplantar injection of IL-6 enhanced the expression of BDNF in the L4-L6 DRG and that this effect was blocked by popliteal fossa injection of ciliobrevin D (Figure 2D). These results link retrograde axonal transport in the sciatic nerve to IL-6-mediated changes in BDNF expression in the DRG, however the experiment cannot specify CREB as a regulator for the IL-6-mediated effects. To address this, we took advantage of CREB binding to cAMP response element (CRE) consensus sequences in DNA, an event normally linked to CREB’s action as a transcription factor. To disrupt the binding of CREB to CREs we administered intrathecal DNA decoy oligonucleotides that contain either the CRE consensus or mutant sequence [18-20]. The decoy CRE consensus oligonucleotides prevented the development of i.pl. IL-6-mediated mechanical hypersensitivity and hyperalgesic priming whereas CRE mutant oligonucleotides were without effect (Figure 3A). Moreover, i.t. administration of the CRE consensus decoy oligonucleotides, but not mutant oligonucleotides, prevented IL-6-mediated increases in BDNF expression in DRG 3 hrs after IL-6 treament (Figure 3B). These findings link IL-6-mediated enhanced expression of BNDF in the DRG and IL-6-induced mechanical hypersensitivity and hyperalgesic priming to the retrograde transport of CREB.

**Figure 4:**
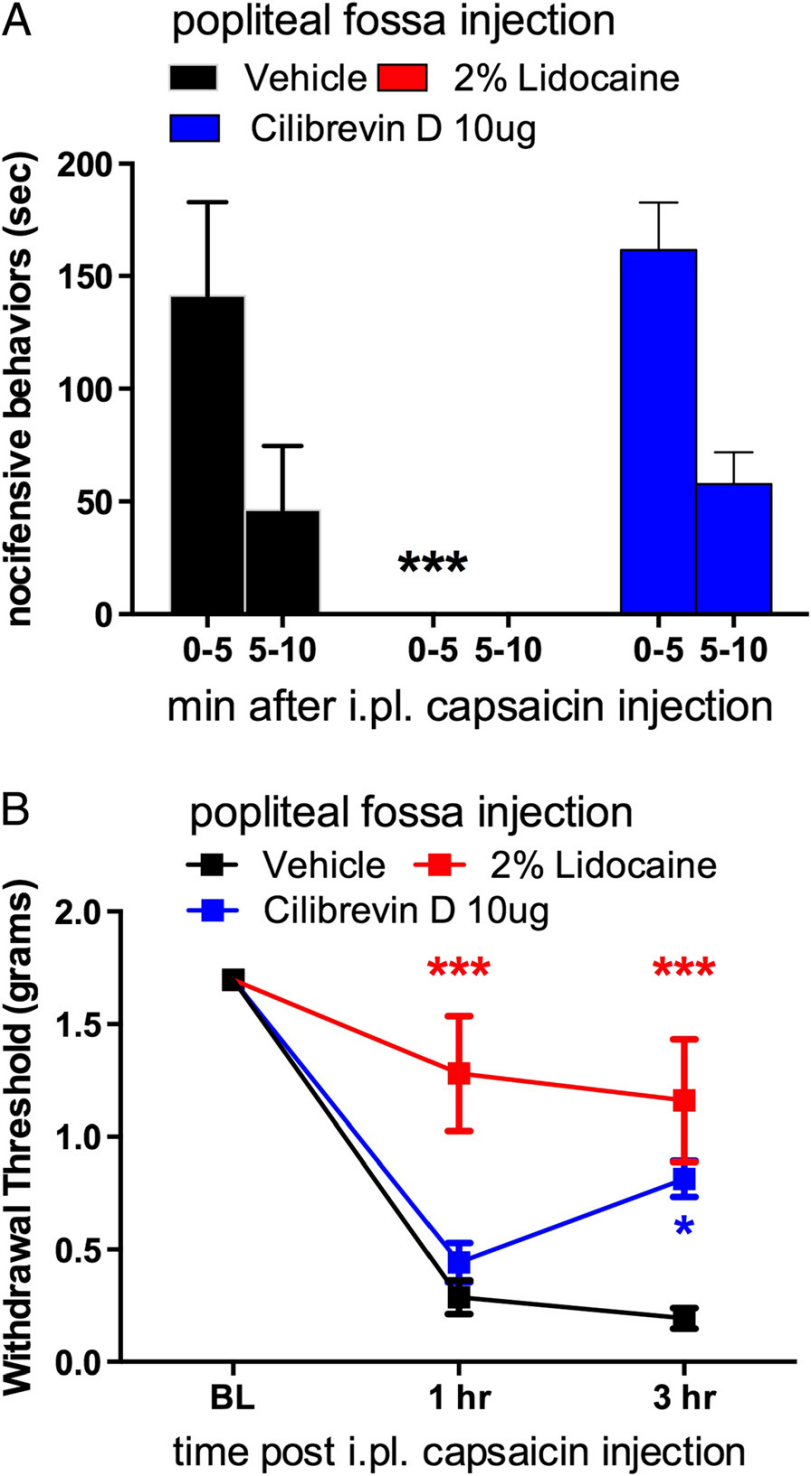
**Popliteal fossa injection of ciliobrevin D does not influence capsaicin-induced flinching but does affect late mechanical hypersensitivity. A)** Popliteal fossa injection of vehicle or ciliobrevin D had no effect on capsaicin (0.9%)-induced nocifensive behaviors whereas lidocaine injection completely blocked the effects of capsaicin. N = 4 - 5 mice per condition. **B)** Lidocaine completely blocked capsaicin-induced mechanical hypersensitivity whereas ciliobrevin D only had an effect at 3 hrs after capsaicin injection. N = 4 – 5 mice per condition. * = p < 0.05 and *** = p < 0.001.

Nociceptive plasticity in the dorsal horn is regulated by numerous transcription factors which include CREB [21,22]. A possible interpretation of the CRE decoy oligonucleotide experiment is blockade of CREB action in dorsal horn neurons. Thus, we aimed to demonstrate that CREB acts upstream of BDNF in DRG neurons. To this end, we co-injected i.t. BDNF, at a dose we previously determined to induce mechanical hypersensitivity and hyperalgesic priming of a similar duration and magnitude to IL-6, with and without CRE consensus decoy oligonucleotides. Unlike experiments where IL-6 was injected into the paw, CRE consensus oligonucleotides failed to alter i.t. BDNF-induced mechanical hypersensitivity or hyperalgesic priming (Figure 3C). Therefore, we conclude that CREB acts upstream of BDNF in the DRG and that CREB is not a key mediator of BDNF-induced nociceptive plasticity in the dorsal horn, at least over this time course.Finally we asked whether the effects of ciliobrevin D might be mediated by effects on nerve conduction. Because IL-6-induced mechanical hypersensitivity develops slowly and there is no acute response to IL-6 we turned to capsaicin in these experiments. Mice received popliteal fossa injections of vehicle, lidocaine (2%) or ciliobrevin D (10 μg) and i.pl. injections of 0.9% capsaicin 5-10 min later. While robust nocifensive responses to capsaicin were observed in the vehicle and ciliobrevin D treated mice, lidocaine, as expected, completely blocked this effect (Figure 4A). We then measured mechanical hypersensitivity in response to capsaicin at 1 and 3 hrs after i.pl. injection. At 1 hr, mechanical hypersensitivity was observed in the vehicle and ciliobrevin D groups but at 3 hrs there was an attenuation of mechanical hypersensitivity in the ciliobrevin D group compared to vehicle (Figure 4B). Mechanical hypersensitivity was completely blocked by lidocaine injection (Figure 4B). These results are consistent with the time course of retrograde transport in IL-6 experiments with ciliobrevin D and demonstrate that ciliobrevin D has no effect on acute pain-related behaviors or mechanical hypersensitivity.

## Discussion

CREB is a major transcription factor that mediates activity-dependent gene transcription across a wide variety of cell types [23]. CREB is a crucial component of transcriptional enhancers that regulate functions of the developing and mature brain, including in neuronal survival, synaptogenesis, synaptic plasticity and drug addiction [22,24-26]. CREB has also been demonstrated to be crucial for the development of mechanical and thermal hypersensitivity in a variety of preclinical pain models [27-31]. However, post-translational mechanisms of CREB regulation (e.g. phosphorylation) or CREB DNA targets have been the focus of those studies [27,32-36]. Our current results add a new layer of evidence to the role of CREB in nociceptive plasticity and uncover a new mechanism for the development of chronic pain – local translation and retrograde transport of a transcription factor.

Our findings support a model wherein IL-6-mediated mechanical sensitization is mediated by the local translation of CREB, its retrograde transport and subsequent induction of BDNF expression in the DRG. Evidence for this model is three pronged. 1) IL-6 treatment leads to nascent synthesis of CREB and the de novo presence of augmented nascently synthesized CREB in the distal sciatic nerve. 2) Disruption of axonal transport prevents the development of mechanical hypersensitivity and hyperalgesic priming and prevents increased BDNF expression in the DRG. 3) Employment of i.t. delivered, CRE decoy oligonucleotides demonstrates that the IL-6-dependent change in BDNF expression and nociceptive plasticity are selectively dependent on CREB within the DRG. Hence, these findings demonstrate a novel mechanism linking locally generated signals in the form of the nascent synthesis of a transcription factor within the peripheral axons of sensory neurons to remote molecular effects within the cell bodies that manifest as altered mechanical hypersensitivity and hyperalgesic priming.

BDNF is well recognized as an important player in pain sensitization [14,37-39]. Previous studies have demonstrated that nociceptor-specific knockout of BDNF leads to a loss of pain sensitization across inflammatory pain models [38]. Moreover, hindpaw inflammation, hindpaw treatment with NGF [40] or DRG neuron treatment *in vitro* with NGF [41] increases BDNF mRNA expression. BDNF mRNA is heavily alternatively spliced with 8 possible 5’ untranslated region (UTR) exons spliced to exon 9 which contains the coding sequence. Inflammation increases expression of several exons but the effect is most robust for exon 1 [41], which is known to respond to CREB-dependent promoters [17]. Here we observed an increase in BDNF protein in DRG after hindpaw IL-6 treatment that was dependent on retrograde transport via the sciatic nerve and that was blocked by i.t. treatment with CRE decoy oligonucleotides. A possible alternative interpretation is that i.t. injection of CRE decoys attenuates CREB signaling in the dorsal horn of the spinal cord. Our finding that CRE decoy treatment fails to alleviate i.t. BDNF-induced mechanical hypersensitivity argues against a dorsal horn mediated effect pointing to a DRG-dependent mechanism. We have previously demonstrated a key role for BDNF in the initiation and maintenance of IL-6-induced hyperalgesic priming [14,42]. We have also shown a critical role for local translation in the initiation but not maintenance of IL-6-induced hyperalgesic priming [6]. Several other studies have demonstrated similar links between translation control in DRG neurons and hyperalgesic priming [43-45]. Our present findings present a possible unification between these two mechanisms in the form of locally translated CREB at the time of IL-6 injection linking the initiation and maintenance of hyperalgesic priming to CRE-dependent changes in BDNF expression in the DRG. Future studies will be needed to evaluate the time course of altered BDNF expression in hyperalgesic priming and whether BDNF dependency during the maintenance phase requires nociceptor-derived BDNF or if it originates from other cells, such as microglia [46-48].

Our results are consistent with a previous study linking axonal translation of CREB in embryonic DRG neurons to NGF-induced survival [15]. This study demonstrated the presence of CREB mRNA in DRG axons and induction of local CREB translation upon NGF exposure. This nascently synthesized CREB then undergoes retrograde transport and influences CRE-dependent transcription in sensory neuron nuclei. Interestingly, this retrograde transport of axonally synthesized CREB was required to account for nuclear accumulation of phosphorylated CREB despite the uninterrupted presence of retrograde signaling endosomes. This suggests that local translation, not retrograde endosomal signaling, constitutes the necessary machinery for CRE-dependent changes in transcription via axonal signaling. In development this locally synthesized, retrograde signal is important for NGF-dependent survival of sensory neurons [15]. In adulthood our results suggest that this same mechanism is used to link local insult to changes in nociceptive sensitivity potentially contributing to the development of a chronic pain state. Although axonal CREB transcripts have been observed in DRG neurons [32], similar studies in sympathetic neurons have failed to detect axonal CREB transcripts [49]. At this point it is not clear if axonal mRNA sorting for CREB transcripts is an exclusive feature of sensory axons or if sympathetic axons are an exception. CREB is a critical mediator of neuronal plasticity in *Aplysia californica*[1,50], an organism where axon crush creates hyperexcitability in a manner reminiscent of observations in neuropathic pain models and patients [1,4,8]. Interestingly, this effect is mediated by local translation [4] and involves retrograde transport of an unidentified signaling protein [8]. Hence, the mechanism we describe here has striking similarity to sensory neuron plasticity in this phylogenetically distinct organism and suggests an evolutionarily conserved mechanism of nociceptive plasticity.

The time course of effects noted in this study deserves comment in relation to measurements of axonal transport. We estimate the distance between the axonal terminals in the hindpaw of mice used in this study to the DRG cell body at ~ 45 mm. At the upper end of estimations for retrograde transport it would take ~ 2.5 hrs for axonally synthesized CREB to reach the DRG nucleus [51,52]. A previous study in embryonic DRG neurons in culture observed retrograde transport rates of ~ 8-9 mm/hr for axonally synthesized CREB labeled with the photoactivatable florescent probe Dendra [15]. We observed robustly increased nascently synthesized CREB (AHA-labelled) in the distal sciatic nerve 2 hrs following intraplantar injection of IL-6. Moreover, we noted an increase in BDNF protein in the DRG, which was reversed by either blockade of retrograde transport or by CRE DNA decoy treatment [18-20], within 3 hrs of IL-6 treatment. These effects are within the time frame permissible from previous measures of retrograde transport [51,52] but are slightly slower than observations made with photactivatable CREB in embryonic DRG neurons *in vitro*[15]. A very recent study indicated that prior injury can increase anterograde and retrograde transport rates in DRG neurons *in vivo* and *in vitro*[53]. While this study used prior nerve crush as the conditioning stimulus, it is interesting to speculate that inflammatory stimuli, such as IL-6, may be able to induce plasticity in axonal transport rates.

There are several caveats to our work that should be addressed. First, the i.t. approach to the CRE DNA decoy experiment can be mediated either by an effect on DRG neurons or spinal cord neurons. CREB has been linked to pain plasticity at both of these locations. As mentioned above, BNDF given i.t. at the same time as the CRE DNA decoy treatment failed to modulate BDNF-induced pain plasticity. This is despite the previous demonstration that BDNF induces CREB phosphorylation in dorsal horn neurons [29]. Hence, we favor an interpretation of our findings wherein CRE DNA decoy treatment is disrupting CREB-mediated actions in DRG neurons that are linked to changes in BDNF expression. We favor a role for retrograde transport of CREB in this effect as blocking retrograde transport attenuated the induction of BDNF in the DRG, however, it is formally possible that this effect is mediated by retrograde signaling endosomes that stimulate CREB activity in the DRG. Ultimately, specific knockdown of axonal CREB mRNA will be needed to provide definitive proof for this model. Second, the compounds used here to disrupt axonal transport have influences on other cells. We cannot completely exclude an inhibitory effect on local inflammation, for instance, within the nerve in response to IL-6 treatment, however, such an effect seems unlikely given that IL-6 treatment itself does not cause any noticeable swelling or flare at the site of injection. We can exclude a potential systemic effect of these compounds based on experiments where they were injected into the contralateral leg. It is also not likely that these transport inhibitors interfere with nerve conduction as ciliobrevin D had no effect on capsaicin-induced flinching while this was completely blocked by lidocaine. Finally, our interpretation of protein localization of CREB and AHA-incorporated CREB in the distal sciatic nerve after IL-6 injection is consistent with retrograde transport, however, we cannot completely rule out a possible contribution of other cells. Having said this, the sciatic nerve was taken 2 cm from the site of AHA injection and it is unlikely that sufficient AHA would travel this distance and incorporate into CREB in sufficient levels for detection using the methods employed here. Moreover, even if this contributed to our observations, such a local effect is completely incompatible with our findings linking retrograde transport and CREB-mediated effects in the DRG to behavioral responses induced by IL-6. Therefore, despite these caveats, we propose that the current evidence favors our model and excludes other alternatives.

We have demonstrated a novel mechanism of nociceptive plasticity linking local translation to changes in gene expression in the distal DRG. Translation control in DRG neurons has emerged as an important mechanism of pain plasticity [5,7,54] but, to our knowledge, no studies have definitively linked a specific locally translated protein to functional changes in the nociceptive system. CREB, an evolutionarily conserved gene controlling neural plasticity [50,55], emerges from our work as a first candidate in this category.

## Materials and methods

### Behavioral testing

Male ICR mice (Harlan, 20-25 g) were used. All animal procedures were approved by the Institutional Animal Care and Use Committee of The University of Arizona and were in accordance with International Association for the Study of Pain and National Institutes of Health Animal Care guidelines. Animals were placed in acrylic boxes with wire mesh floors and allowed to habituate for 1 hour prior to all behavioral testing. The experimenter making measurements was always blinded to the experimental conditions. IL-6 (0.1 ng) was injected into the plantar surface of the left hindpaw in a volume of 25 μl. BDNF was injected intrathecally (i.t.) in a volume of 5 μl. Popliteal fossa injections were done immediately after intraplantar (i.pl.) injections under brief (<3 min) isoflurane anesthesia in a volume of 50 μl. The exception was i.pl. capsaicin experiments where the popliteal fossa injection was done 5-10 min prior to the capsaicin injection. For i.t. treatments compounds were injected immediately after i.pl. injections under brief (<3 min) isoflurane anesthesia in a volume of 5 μl. PGE_2_ (100 ng) was injected i.pl. on day 6 or later in a volume of 25 μl. Mechanical testing was done using the up-down method at time points indicated in the text. Nocifensive behavior induced by capsaicin injection was observed over 15 min in mechanical testing boxes following habituation of the animals.

### Primary neuronal cultures

DRG from ICR mice were excised aseptically and placed in Hank's Buffered Salt Solution (HBSS, Invitrogen, Grand Island, NY) on ice. The ganglia were dissociated enzymatically with collagenase A (1 mg/ml, 25 min, Roche, Indianapolis, IN) and collagenase D (1 mg/ml, Roche, Indianapolis, IN) with papain (30 units/ml) for 20 min at 37°C. To eliminate debris, 70 μm (BD Biosciences, San Jose, CA) cell strainers were used. The dissociated cells were resuspended in DMEM/F12 (Invitrogen, Grand Island, NY) containing 1X pen-strep (Invitrogen, Grand Island, NY), 1X GlutaMax, 3 μg/ml 5-FDU (Sigma, St. Louis, MO), 7 μg/ml uridine (Sigma, St. Louis, MO) and 10% fetal bovine serum (Thermo Hyclone, Rockford, IL). The cells were plated in 6-well plates (BD Biosciences) and incubated at 37°C in a humidified 95% air/5%CO_2_ incubator. On day 5 the cells were washed in DMEM/F12 media for 30 min followed by treatment with escalating doses of IL-6 for 15 minutes.

### Nascent CREB synthesis assay

Azidohomoalanine (AHA) is a methionine analogue that cells can incorporate into nascentlly synthesized proteins. The left paws of ICR mice were injected with vehicle or IL-6 (0.1 ng) along with AHA (300 ng, Life technologies) in 25 μl sterile phosphate buffered saline (PBS). After 15 min or 2 hrs sciatic nerves, taken 2 cm from the injection site, were harvested. Protein was extracted from the nerves by ultrasonication, centrifugation at 20000 × g for 15 min and collection of the supernatant. Equal amount of protein was used to immunoprecipitate CREB by incubating the supernatant with 1:50 mouse anti-CREB antibody conjugated to Sepharose beads (cat # 3955, Cell Signalling, Danvers, MA) overnight at 4°C. This was followed by centrifugation to precipitate the beads. The pelleted beads were suspended in Tris-SDS buffer (1% SDS and 50 mM Tris-HCl, pH 8.0), centrifuged and the supernatant was collected. At this stage, the supernatant contains the immunoprecipitated CREB where the nascently synthesized form would have incorporated AHA. AHA was biotinylated using Click-it Protein Analysis Detection Kit (Life technologies, Grand Island, NY) according to the manufacturer’s instructions. The biotinylated nascently synthesized CREB was detected by Western blotting.

### Western blotting

Protein was extracted from the cells and tissue in lysis buffer (50 mM Tris HCl, 1% Triton X-100, 150 mM NaCl, and 1 mM EDTA at pH 7.4) containing protease and phosphatase inhibitor mixtures (Sigma, St. Louis, MO) with an ultrasonicator on ice, and cleared of cellular debris and nuclei by centrifugation at 14,000 RCF for 15 min at 4°C. Fifteen micrograms of protein per well were loaded and separated by standard 7.5% or 10% SDS-PAGE. Proteins were transferred to Immobilon-P membranes (Millipore, Billerica, MA) and then blocked with 5% dry milk for 3 h at room temperature. The blots were incubated with primary antibody overnight at 4°C and detected the following day with donkey anti-rabbit antibody conjugated to horseradish peroxidase (Jackson Immunoresearch, West Grove, PA). Signal was detected by ECL on chemiluminescent films. Densitometric analyses were performed with Image J software (NIH, Bethesda, MD).

### Drugs and primary antibodies

The anti-CREB rabbit antibodies (cat# 9197) were obtained from Cell Signaling (Danvers, MA). The BDNF antibody was from Sigma (cat# AV41970, St. Louis, MO) and βIII-Tubulin was from Promega (cat# G7121, Madison, WI). The CRE concensus (cat# sc-2504) and mutant (cat# sc-2517) oligonucleotides were from Santa Cruz Biotechnologies (Santa Cruz, CA). Human recombinant IL-6 and BDNF were from R&D Systems. Colchicine and nocodazole were from Tocris Bioscience; Ciliobrevin D was from EMD Millipore and PGE_2_ was from Cayman Chemical Company. Capsaicin and lidocaine were from Sigma Aldrich. Stock solutions for colchicine, nocodazole and ciliobrevin D were made in cell culture grade 100% DMSO. BDNF and IL-6 stock solution was made in sterile PBS containing 0.1% BSA and TrkB/Fc stock solution was made in sterile PBS. Capsaicin and PGE_2_ stock solutions were made in 100% ethanol. Lidocaine stock solutions were made in sterile saline. All drugs except were diluted to final concentrations in sterile PBS for injection.

### Statistical analysis and data presentation

Data are shown as means and the standard error of the mean (±SEM) of *n* = 8 independent cell culture wells, *n* = 6 tissue samples for *in vivo* Western blotting, and immunoprecipitation or *n* = 6 independent animals for behavioral studies. Graph plotting and statistical analysis used GraphPad Prism Version 5.03 (Graph Pad Software, Inc. San Diego, CA, USA). Statistical evaluation was performed by one- or two-way analysis of variance (ANOVA), followed, where appropriate, by Dunnet post-hoc analysis for behavioral and Western blot data. Where only two comparisons were made (Figure 1B and C) student’s t-test was used. The *a priori* level of significance was considered to be p < 0.05.

## Competing interests

The authors declare that they have no competing interests.

## Authors’ contributions

OKM designed experiments, analyzed data, participated in writing the manuscript and conducted the biochemical and some of the behavioral experiments. DVT, JKM and MNA conducted behavioral experiments and analyzed data. EKM and SG participated in experimental design. GD designed experiments and participated in experimental design and writing of the manuscript. TJP supervised the project, designed experiments, analyzed data, performed behavioral and biochemical experiments and wrote the manuscript. All authors read and approved the final version of the manuscript.
